# Blakiston’s Visiting List for 1892

**Published:** 1892-01

**Authors:** 


BLAKISTON'S VISITING LIST for 1892 is an elegant, compact, convenient, useful companion.

It contains:—

Hall's Method in Asphyxia, Poisons and Antidotes, Metric System, Dose Table, New Remedies, Aids to Diagnosis and Treatment of Diseases of the Eye, Diagram of Eruption of Milk Teeth, Posological tables, Disinfectants, Examination of Urine, Incompatibility, Table to Calculate Period of Utero-Gestation, Sylvester's Method of Artificial Respiration, Transportation of Injured Persons and Diagram of Chest.

It also contains the blanks usually found in first-class visiting lists.

It is issued in Regular Edition, Inter-Leaved Edition. Perpetual Edition same a Regular Edition without dates.

Published by Blakiston, Son & Co., Philadelphia. Sent on receipt of price.

---

### THE COLUMBIA DAILY CALENDAR.

An old friend in a new dress, and an article that has come to be one of the indispensables of an editor's desk, comes to hand in the Columbia Daily Calendar for 1892. The Calend r is in the form of a pad containing 367 leaves, each 5⅛x 2⅝ inches; and each slip bears a short paragraph pertaining to cycling or some kindred subject, and at the bottom of each leaf is a blank for memnranda. The stand is an entirely new departure, being made of sheet metal finished in ivory black, and is very compact. This is the seventh issue of this now well-known Calendar, yet all the matter is fresh and new.

